# Metabolomic Analysis of Renal Cell Carcinoma in the Prostate, Lung, Colorectal, and Ovarian Cancer Screening Trial

**DOI:** 10.3390/metabo12121189

**Published:** 2022-11-29

**Authors:** Kathleen M. McClain, Joshua N. Sampson, Jessica L. Petrick, Kaitlyn M. Mazzilli, Robert E. Gerszten, Clary B. Clish, Mark P. Purdue, Loren Lipworth, Steven C. Moore

**Affiliations:** 1 Division of Cancer Epidemiology and Genetics, National Cancer Institute, Bethesda, MD 20892, USA; 2 Slone Epidemiology Center at Boston University, Boston, MA 02118, USA; 3 Division of Cardiovascular Medicine, Beth Israel Deaconess Medical Center, Boston, MA 02215, USA; 4 Broad Institute of MIT and Harvard, Cambridge, MA 02142, USA; 5 Division of Epidemiology, Department of Medicine, Vanderbilt University Medical Center, Nashville, TN 37232, USA

**Keywords:** metabolomics, kidney cancer, renal cell carcinoma

## Abstract

**Background:** In the US in 2021, 76,080 kidney cancers are expected and >80% are renal cell carcinomas (RCCs). Along with excess fat, metabolic dysfunction is implicated in RCC etiology. To identify RCC-associated metabolites, we conducted a 1:1 matched case–control study nested within the Prostate, Lung, Colorectal, and Ovarian (PLCO) Cancer Screening Trial. **Methods:** We measured 522 serum metabolites in 267 cases/control pairs. Cases were followed for a median 7.1 years from blood draw to diagnosis. Using conditional logistic regression, we computed adjusted odds ratios (ORs) and 95% confidence intervals (CIs) comparing risk between 90th and 10th percentiles of log metabolite intensity, with the significance threshold at a false discovery rate <0.20. **Results:** Four metabolites were inversely associated with risk of RCC during follow-up—C38:4 PI, C34:0 PC, C14:0 SM, and C16:1 SM (ORs ranging from 0.33–0.44). Two were positively associated with RCC risk—C3-DC-CH3 carnitine and C5 carnitine (ORs = 2.84 and 2.83, respectively). These results were robust when further adjusted for metabolic risk factors (body mass index (BMI), physical activity, diabetes/hypertension history). Metabolites associated with RCC had weak correlations (|*r*| < 0.2) with risk factors of BMI, physical activity, smoking, alcohol, and diabetes/hypertension history. In mutually adjusted models, three metabolites (C38:4 PI, C14:0 SM, and C3-DC-CH3 carnitine) were independently associated with RCC risk. **Conclusions:** Serum concentrations of six metabolites were associated with RCC risk, and three of these had independent associations from the mutually adjusted model. These metabolites may point toward new biological pathways of relevance to this malignancy.

## 1. Introduction

An estimated 79,000 new cases of kidney cancer are expected in the United States (US) in 2022, making it the eighth most commonly diagnosed primary cancer [1]. Approximately 80–90% of kidney cancers are renal cell carcinomas (RCC) [2].

A hallmark of RCC is the major role played by perturbed metabolism in its etiology. Metabolic risk factors such as excess body fatness and hypertension have strong, well-established associations with the development of RCC (i.e., RCC risk) [3,4,5]. Heritable conditions that increase RCC risk often have a metabolic component, such as the change in hypoxia response in Von Hippel–Landau disease [6] and the production of fumarase (part of the TCA cycle) in hereditary leiomyomatosis [7]. In RCC tumors, metabolism is shifted toward increased glucose and lactate production, consistent with Warburg metabolism [8,9,10]. Metabolic flux through glycolysis also appears to be “partitioned”, with a high production of early glycolysis intermediates accompanied by low production of late-stage metabolites [10,11]. The early-stage intermediates are diverted toward the pentose phosphate pathway, which promotes anabolic reactions and redox homeostasis, while later-stage phosphates are diverted toward TCA and one-carbon metabolism [8,10,11]. Numerous changes in TCA metabolism suggest that mitochondrial bioenergetics and oxidative phosphorylation processes are also impaired in RCC, and an intracellular accumulation of fatty acids suggests an enhanced uptake and synthesis of fatty acids [10,11,12]. These changes in lipid metabolism may in turn relate to increased levels of acylcarnitines [13] and changes in glycerol enzymes [14].

Whether changes in systemic circulating metabolism can be observed prior to RCC carcinogenesis and/or diagnosis is not yet established. Non-prospective studies have used metabolomics to identify associations of RCC risk with levels of metabolites in blood [15,16,17,18], urine [19,20,21,22,23], and tumor tissue [11,13,24]. Their findings suggest that RCC patients have systemic increases in glycolysis and acylcarnitine production and a decrease in TCA oxidative metabolisms in [22,25]. However, only one prospective study, by Guida et al. has been conducted to date, primarily finding changes in glycerophospholipid metabolism [26].

Identifying prospective changes in metabolism is an important next step that can help to establish the sequence of RCC pathogenesis, clarify its etiology, and provide clues about why risk factors such as obesity are linked with risk. Regarding obesity, evidence points toward several possible explanations for its association with RCC risk, including oxidative stress [27], hypertension-induced injury to the renal tubules [28,29], renal atherosclerosis [30], disruption of normal endocrine activity [31,32,33,34], and chronic inflammation [35] but direct assessment of the metabolites that may mediate the obesity–RCC association have been uncommon. Prospective designs also help eliminate prodromal effects of undiagnosed tumors, as well as biases related to case–control selection and sample handling [36]. The Guida et al. study has provided strong initial leads regarding prospective metabolic risk factors for RCC, but further studies are needed to replicate these results and to explore additional metabolites using different platforms.

We, therefore, conducted metabolic profiling of pre-diagnostic sera from a nested case–control study of 267 RCC cases and 267 controls in the Prostate, Lung, Colorectal, and Ovarian (PLCO) Cancer Screening Trial. Our hypothesis was that biomarkers associated with RCC risk can be detected in serum before the overt presentation of the disease. Identification of such biomarkers may help clarify how metabolic factors contribute to the etiology of RCC.

## 2. Methods

### 2.1. Study Population

The PLCO Cancer Screening Trial is a population-based, multi-center, randomized screening trial of >150,000 US men and women aged 55–74 with no history of prostate, lung, colorectal, or ovarian cancers. Participants were assigned to a screening arm or a control arm. The PLCO Cancer Screening Trial was approved by the Institutional Review Boards at the National Cancer Institute and the study centers, and all participants provided informed consent.

PLCO participants included in this nested case–control study were all from the screening arm of the trial. Eligible cases were those with a histologically confirmed incident first primary diagnosis of RCC (International Classification of Disease for Oncology code: ICD-0-3 C64.9) ascertained by medical record review. Cases were followed for a median of 7.1 years (interquartile range (IQR): 4.3–9.7), measured from blood sample collection to date of diagnosis. Controls had no history of RCC and were individually matched to cases by age at baseline, sex, recruitment site, menopausal status (for women), and season and year of blood draw. A total of 267 RCC cases and 267 controls were identified for our analyses.

### 2.2. Metabolomic Profiling

A total of 531 metabolites were measured in non-fasting serum samples collected at the first PLCO screening arm follow-up visit. The samples had been processed within two hours of collection and stored at −70 °C. Serum vials never previously thawed were selected for this project. The metabolomics data were generated by the lab of Clary Clish at the Broad Institute and the lab of Dr. Robert Gerszten, then at Massachusetts General Hospital. Methodologies for both the Broad Institute [37] and the Gerszten lab [38] have been published previously and are described in brief below (full detail available in Supplementary Table S1).

From the lab of Dr. Clish, metabolites were measured with two liquid chromatography (LC)–mass spectroscopy (MS) methods. Lipids were analyzed using a Nexera X2 U-HPLC (Shimadzu, Marlborough, MA, USA) coupled with an Exactive Plus Orbitrap mass spectrometer (Thermo Fisher Scientific, Waltham, MA, USA). Hydrophilic interaction liquid chromatography analyses of water-soluble metabolites were conducted in the positive-ion mode using a Nexera X2 U-HPLC (Shimadzu, Marlborough, MA, USA)-Q Exactive Orbitrap (Thermo Fisher Scientific; Waltham, MA, USA) LC–MS instrument. From the lab of Dr. Gerszten, metabolites were measured using the high sensitivity Agilent 6490 QQQ MS (Agilent Technologies, Inc., Santa Clara, CA, USA) in the negative ion mode via multiple reaction monitoring scanning. The Clish lab quantified levels of 462 metabolites and the Gerszten lab 69 metabolites. Nine metabolites were measured by both labs (allantoin, bilirubin, citrulline, hypoxanthine, inosine, kynurenic acid, taurine, xanthine, and xanthosine), and we retained the Clish lab metabolite levels for those nine overlapping metabolites.

Metabolite levels with values below the limit of detection were assigned half the minimum observed value for that metabolite. Metabolite levels were natural log-normalized for analyses. Intraclass correlation coefficients (ICCs) were determined from 40 quality control samples interspersed across batches. The median (IQR) ICC across the 522 included metabolites was 0.97 (0.92–0.99).

### 2.3. Covariate Assessment

Covariate information, including established and suspected RCC risk factors, was obtained through questionnaires completed by participants at trial baseline. Potential covariates included: age (years), sex (male, female), race/ethnicity (self-reported non-Hispanic White, non-Hispanic Black, other race/ethnicity), body mass index (BMI; kg/m^2^), physical activity (none, <1 h/week, 1 h/week, 2 h/week, 3 h/week, 4+ h/week), history of diabetes (yes, no), history of hypertension (yes, no), cigarette smoking status (never, former, current), alcohol consumption (g/day), and family history of renal cancer (yes, no, unsure). For alcohol consumption, 26 participants had missing values. In order to retain these observations, we imputed the median alcohol intake value, completed separately for cases and controls. Similarly, for physical activity, we modeled the non-response using missing indicator variables.

### 2.4. Statistical Analyses

Conditional logistic regression models were used to estimate odds ratios (ORs) and 95% confidence intervals (CIs) for the associations between metabolites and RCC risk during follow-up, where the ORs correspond to risk at the 90th percentile compared to the 10th percentile of log metabolite intensity. Models were sequentially adjusted for the following factors: (1) age, sex, race/ethnicity, cigarette smoking status, alcohol consumption, and family history of renal cancer, and (2) BMI, physical activity, history of diabetes, and history of hypertension. The threshold for statistical significance was set at a false discovery rate of 0.20, the threshold used in several prior prospective metabolomics analyses [39,40,41].

In order to examine potential independence of observed associations, we used a forward selection approach to create a mutually adjusted model. Specifically, we modeled each metabolite in relation to RCC, retained the metabolite with the lowest *p*-value, and modeled the remaining metabolites, repeating this process until reaching the false discovery threshold. To assess whether metabolite–RCC associations may relate to other RCC risk factors, we estimated correlations between significant metabolites and covariates included in our model 2.

We evaluated if the relationships between metabolites that were significant in model 2 and RCC risk were non-linear using restricted cubic splines. For each metabolite–RCC association, the *p* for curvature exceeded 0.05 (Supplementary Table S2), suggesting associations are linear. Consequently, the results presented are based on linear functions.

In exploratory mediation analyses, we decomposed the total effect of BMI on RCC into an indirect effect through metabolites and a direct effect through other pathways [42]. We estimated the total effect of BMI on RCC (OR_total effect_) using conditional logistic models adjusted for age, sex, race/ethnicity, cigarette smoking status, alcohol consumption, family history of renal cancer, history of diabetes, and history of hypertension. The direct effect of BMI on RCC through non-metabolite pathways (OR_direct effect_) was estimated using conditional logistic models adjusted for the covariates mentioned above along with metabolites. We estimated the indirect effect of BMI on RCC through metabolites (OR_indirect effect_) as OR_total effect_/OR_direct effect_. The attenuation of ORs was defined as [log(OR_total effect_)−log(OR_direct effect_)]/log(OR_total effect_).

We also conducted two sets of sensitivity analyses. The first was removing cases diagnosed within the first two years of study (*n* = 29) to assess if any of the associations observed could have been influenced by those potentially latent RCCs. The second sensitivity analysis was examining metabolite–RCC associations stratified by time between blood draw and diagnosis. For these analyses we cut at the median time on the study for cases, which was 7.10 years (IQR = 4.34–9.68). We then calculated *p*-values for the interactions between metabolites and median time on study, using the Bonferroni-adjusted *p*-value = 9.58 × 10^−5^ (=0.05/522).

Analyses were performed using SAS 9.4 (SAS Institute Inc., Cary, NC, USA).

## 3. Results

The Sociodemographic, lifestyle, and medical history characteristics of the 534 participants are presented by case–control status in Table 1. The participants were, on average, 63 years old, and primarily male and White. BMI was the only characteristic that had a statistically significant difference (*p* < 0.05) between cases and controls, with a higher proportion of cases classified as obese.

When adjusting for non-metabolic RCC risk factors, 82 metabolites were significantly associated with RCC risk at the false discovery rate < 0.20 (Supplementary Table S3). Further adjustment for metabolic risk factors (BMI, physical activity, history of diabetes, and history of hypertension) reduced the number of metabolites significantly associated with RCC to six (Table 2). These six metabolites included two glycerophospholipids (C38:4 PI and C34:0 PC) inversely associated with risk, two acylcarnitines (C3-DC-CH3 carnitine and C5 carnitine) positively associated with risk, one sphingolipid inversely associated with risk (C14:0 SM) and one organic nitrogen compound inversely associated with risk (C16:1 SM). The respective odds ratios comparing risk at the 90th vs. 10th percentiles for these were 0.32 (95%CI: 0.18–0.58), 0.43 (0.26–0.74), 2.61 (1.53–4.47), 2.31 (1.36–3.93), 0.40 (0.24–0.68), 0.34 (0.19–0.63).

Because we were primarily interested in identifying mediators, we prioritized metabolites with independent effects on RCC risk. Correlations between metabolites and RCC risk factors were weak, with |*r*| < 0.20 (excluding metabolite–metabolite correlations of similar pathways; Supplementary Table S4), suggesting these associations were independent of other risk factors. In our mutual adjustment model (Table 3), three of the metabolites were retained (C38:4 PI, C14:0 SM, C3-DC-CH3 carnitine) with slight attenuation of ORs.

The effect of BMI was not meaningfully changed when adding metabolites as potential mediators (Table 4). The OR for BMI was strengthened when adding four of the metabolites (ranging from −3.8% to −15.7%) and was attenuated when adding the two acylcarnitines (19.7% and 22.4%). Similarly, adding BMI to the models had a minor effect on metabolites–RCC associations (Table 5); carnitine associations were attenuated (12.3% and 16.8%), while the other metabolite classes’ associations were strengthened (ranging from −5.6% to −21.0%).

Regarding our sensitivity analyses, removing cases diagnosed within the first two years of the study negligibly changed metabolite–RCC associations (Supplementary Data). Similarly, there was no evidence of interaction between metabolites and median time in the study as no *p*-values were <9.58 × 10^−5^ (Supplementary Data).

## 4. Discussion

In this nested case–control study from the PLCO Cancer Screening Trial, 82 metabolites were associated with risk of RCC (at the false discovery rate <0.20) in initial models, and six of these were associated with RCC even after adjusting for BMI, physical activity, history of diabetes, and history of hypertension. We further found that, in mutually adjusted models, three of the metabolites were independently associated with RCC. These three associations have never before been reported in relation to RCC (C38:4 PI, C3-DC-CH3 carnitine, C14:0 SM), and thus constitute novel findings. Because the study is prospective, the associations likely reflect an etiologic role in RCC, rather than prodromal effects of the tumor itself, and are less likely to be influenced by selection and/or sample handling biases than associations from non-prospective studies. We also examined the potential for these metabolites to mediate the obesity–RCC association but found little evidence for such mediation at present. These findings related to specific glycerophospholipids, acylcarnitines, sphingolipids, and organic nitrogen compounds point toward potentially important pathways in the etiology of RCC.

**Figure 1:**
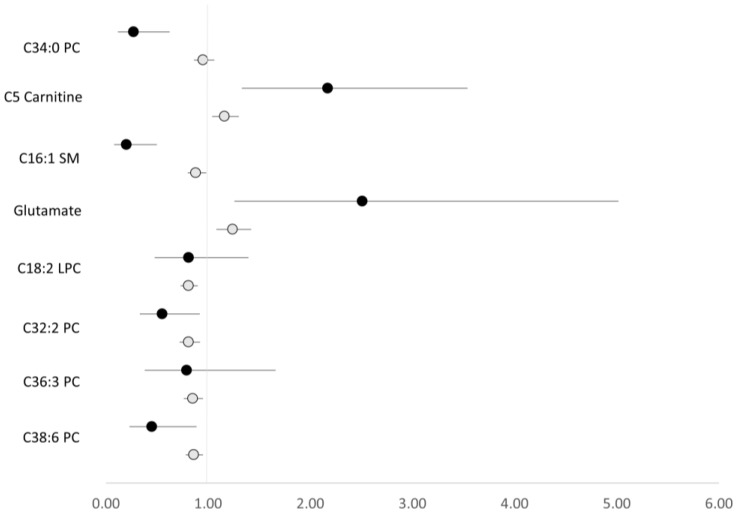
Odds ratios and 95% confidence intervals for RCC per 1 standard deviation (natural log scale) increment in metabolite level, comparing results from PLCO and Guida et al. [24]. Black-filled circles correspond to the PLCO, and gray-filled circles correspond to Guida et al. For reference, the metabolite name abbreviations used above correspond to the abbreviations of PC ae C34:0, isovalerylcarnitine (C5), SM C16:1, glutamate, lysoPC a C18:2, PC ae C32:2, PC ae C36:3, PC ae 38:6 in Guida et al. For the first three metabolites, Guida et al. results are based on crude models in the supplement (only these results were available), while the remainder are based on BMI-adjusted models in the main results.

To our knowledge, ours is the second prospective metabolomics analysis of RCC, the first being an analysis by Guida et al. of 1305 case–control pairs in a European and Australian consortium [26]. This analysis identified 25 metabolites associated with RCC risk, most of which were glycerophospholipids (*n* = 14) and amino acids (*n* = 9). Guida et al. used different platforms than did our study and so only eight RCC-associated metabolites were measured in common between them. Associations for these eight metabolites did not replicate between studies at the multiple testing threshold of statistical significance. Associations did, however, replicate at the nominal level of significance (*p* < 0.05) for five of eight metabolites (C32:2 PC, C38:6 PC, C5 carnitine, C16:1 SM, glutamate), and the direction of effect was the same for all eight (Figure 1). This consistency of findings suggests a reasonably high level of replicability. Non-prospective studies have also examined associations with RCC risk [11,13,15,16,17,18,19,20,21,22,23,24] and some results parallel our own, particularly results related to glycerophospholipids [16,17,20], sphingolipids [16,20], and acylcarnitines [13,16,19].

The exact biology underlying these associations is not fully understood, though basic research suggests several possibilities. Glycerophospholipids are the primary constituent of cell membranes and are key regulators of cell signaling. Prior studies suggest that clear cell RCC cells exhibit increased uptake of glycerophospholipids from circulation, possibly to support growth needs [10,11]. Possibly, increased uptake of glycerophospholipids by incipient tumors during the preclinical stage could explain the low circulating levels we observed. Acylcarnitines are required for the transport of fatty acids into mitochondria and elevated levels in circulation parallel elevated acylcarnitine levels observed in tumors themselves [19]. Increased levels may occur in response to metabolic changes that accompany carcinogenesis, such as reduced fatty acid β-oxidation [19] and/or impaired mitochondrial bioenergetics [10,11,12]. Sphingolipids are structural molecules of cell membranes and signaling molecules that help regulate cell growth, proliferation, migration, and senescence, among other functions. Sphingolipids are a heterogeneous class with respect to their postulated role in carcinogenesis [43], and the role that C14:0 specifically may play is not well-studied or understood. Finally, little is known about the organic nitrogen compound organic C16:1 SM and its role in carcinogenesis therefore remains speculative. Additionally, various sphingolipids, acylcarnitines, and C16:1 SM have been associated with the risk of type 2 diabetes [44,45,46], which may constitute part of the mechanistic pathway linking these metabolites with RCC risk.

Our study has several limitations. First, the PLCO cohort had a limited number of RCC cases, and thus we could only detect associations of a moderate or strong magnitude. Due to the observational nature of this study, we cannot rule out residual confounding by unknown or inadequately measured risk factors. Additionally, PLCO consists predominantly of White US participants whose incidence rates of RCC are lower than those of other demographic groups, such as Black Americans. These associations could be further explored for potential etiological and/or histological subtype differences between White and other racial/ethnic groups in a more diverse study population, for example evidence of Black individuals’ higher incidence of papillary RCC [47]. Future studies should aim to replicate our findings and assess whether they generalize to other at-risk populations and to specific histological subtypes (which could not examine due to limited sample size). Another potential residual confounder of interest are occupational exposures (such as benzene [48], trichloroethylene [49], etc.), which we were not able to assess in our study. Our analysis did not assess all human metabolites, of which there are more than 100,000 [50], but rather the 522 metabolites measured by the Clish and Gerszten platforms. As the sensitivity of MS technologies improves, future studies will be able to examine many more metabolites. Lastly, while we used a false discovery rate <0.20 to control for multiple testing, some findings nevertheless could be due to chance.

There were several strengths to our study. This study is, to our knowledge, the first prospective study to use metabolomics to evaluate serum metabolites in relation to RCC risk. Our use of pre-diagnostic samples should eliminate, or at least minimize, bias resulting from differential sample handling between cases and controls—a problem that can induce false positives in case–control studies [36]. In addition, since cases were diagnosed a median of seven years after sample collection, the associations we observed are unlikely to reflect the effects of preclinical or undiagnosed disease on metabolite levels, especially given that associations were materially unchanged when removing the cases diagnosed closest to sample collection (i.e., within the first two years of follow-up). We used highly reliable metabolomics platforms that measured >500 metabolites in total. Finally, we used mutually adjusted models which allowed us to potentially identify the metabolites most informative about RCC risk.

## 5. Conclusions

In sum, our results show that pre-diagnostic serum levels of six metabolites were associated with RCC risk and three of these (C38:4 PI, C3-DC-CH3 carnitine, and C14:0 SM) remained significantly associated with RCC in mutually adjusted models. These metabolites may point toward new biological pathways of relevance to RCC risk; particularly findings related to specific glycerophospholipids, acylcarnitines, sphingolipids, and organic nitrogen compounds and their potential implications on the etiology of RCC. Further investigations in larger, more diverse cohorts would help establish these findings, while potentially uncovering further novel metabolite–RCC associations.

## Figures and Tables

**Table 1:** Sociodemographic, lifestyle, and medical history characteristics of the renal cell carcinoma case–control study nested within the Prostate, Lung, Colorectal, and Ovarian Cancer Screening Trial.

Characteristic	Cases (*n* = 267)	Controls (*n* = 267)	*p*-Value
**Age (years), mean ± SD**	63.0 ± 4.98	63.0 ± 4.97	Matched
**Sex, %**			Matched
Male	65.9	65.9	
Female	34.1	34.1	
**Race/ethnicity, %**			0.82
White, non-Hispanic	89.1	89.1	
Black, non-Hispanic	6.4	6.4	
Other *	4.5	4.5	
**Body mass index (kg/m^2^),** **mean ± SD**	28.9 ± 5.10	27.4 ± 4.20	<0.0001
**Body mass index category, %**			0.001
0–18.5 kg/m^2^	0.8	0.8	
18.5–25 kg/m^2^	21.0	28.6	
25–30 kg/m^2^	41.2	48.5	
30+ kg/m^2^	37.1	22.2	
**Physical activity, %**			0.33
None	15.0	13.0	
<1 h/week	23.6	21.0	
1 h/week	13.4	13.4	
2 h/week	15.8	12.7	
3 h/week	10.6	17.4	
4+ h/week	21.7	22.5	
**History of diabetes, %**			
No	88.8	90.9	0.41
Yes	11.2	9.1	
**History of hypertension, %**			0.08
No	54.5	62.0	
Yes	45.5	38.0	
**Cigarette smoking status, %**			0.94
Never	46.1	47.6	
Former	43.8	42.3	
Current	10.1	10.1	
**Alcohol consumption (g/day), mean ± SD**	9.2 ± 21.17	13.2 ± 29.32	0.07
**Family history of renal cancer, %**			0.46
No	93.2	95.5	
Yes	1.5	1.5	
Unsure	5.3	3.0	

Percentages may not sum to 100% due to rounding. * Other includes Hispanic, Asian, Pacific Islander, and American Indian. Missing: age (0), sex (0), race (0), body mass index (continuous; 1), body mass index (categorical; 1), physical activity (27), history of diabetes (2), history of hypertension (2), cigarette smoking status (0), alcohol consumption (0), family history of renal cancer (1).

**Table 2:** Odds ratios (ORs) and 95% confidence intervals (CIs) for renal cell carcinoma when comparing the 90th with the 10th percentile levels of metabolites *.

Metabolite	Model 1 ^†^ OR (95% CI)	Model 2 ^‡^ OR (95% CI)
**Glycerophospholipids**		
C38:4 PI	0.35 (0.21–0.61)	0.32 (0.18–0.58)
C34:0 PC	0.43 (0.26–0.72)	0.43 (0.26–0.74)
**Fatty acyls (acylcarnitines)**		
C3-DC-CH3 Carnitine	2.83 (1.73–4.64)	2.61 (1.53–4.47)
C5 Carnitine	2.88 (1.74–4.76)	2.31 (1.36–3.93)
**Sphingolipids**		
C14:0 SM	0.45 (0.26–0.73)	0.40 (0.24–0.68)
**Organic nitrogen compounds**		
C16:1 SM	0.40 (0.23–0.70)	0.34 (0.19–0.63)

* ORs based on conditional logistic regression models with metabolite percentiles based on the distribution in controls on the log metabolite intensity scale; table includes only metabolites for which the false discovery rate was <0.20. ^†^ Model was adjusted for age, sex, race, cigarette smoking status, alcohol consumption, and family history of renal cancer. ^‡^ Model was adjusted for model 1 covariates, plus body mass index, physical activity, history of diabetes, and history of hypertension.

**Table 3:** Odds ratios (ORs) and 95% confidence intervals (CIs) for renal cell carcinoma when comparing the 90th with the 10th percentile levels of metabolites in forward selection models.

Metabolite	Chemical Class	Order Entered	Model Entry *p* *	Mutually Adjusted OR ^†^ (95% CI)	Mutually Adjusted *p* *
C38:4 PI	Glycerophospholipids	1	<0.0001	0.49 (0.26–0.95)	0.03
C3-DC-CH3 Carnitine	Fatty acyls (acylcarnitine)	2	0.002	2.39 (1.39–4.13)	0.002
C14:0 SM	Sphingolipids	3	0.02	0.52 (0.29–0.92)	0.02

* *p*-value for χ^2^ test obtained from conditional logistic regression model for a given metabolite (modeled on a continuous basis); all tests were two-sided. ^†^ ORs correspond to RCC risk at the 90th percentile compared to the 10th of log metabolite intensity; model was adjusted for age, sex, race, body mass index, cigarette smoking status, alcohol consumption, physical activity, family history of renal cancer, history of diabetes, and history of hypertension, and simultaneously adjusted for other listed metabolites (C38:4 PI, C14:0 SM, and C3-DC-CH3 Carnitine only).

**Table 4:** Attenuation of the odds ratio for the BMI-renal cell carcinoma association with the addition (one at a time) of each of the six identified metabolites to the adjustment set.

Metabolite	BMI OR (95% CI) *	Attenuation of Log (OR)
None	1.44 (1.19–1.74)	-
C38:4 PI	1.51 (1.23–1.85)	−13.3%
C34:0 PC	1.46 (1.20–1.77)	−3.8%
C3-DC-CH3 Carnitine	1.34 (1.09–1.64)	19.7%
C5 Carnitine	1.33 (1.08–1.62)	22.4%
C14:0 SM	1.52 (1.24–1.86)	−15.7%
C16:1 SM	1.52 (1.24–1.85)	−14.8%

* ORs correspond to RCC risk with each 5 kg/m^2^ increase in BMI. Model was adjusted for age, sex, race, cigarette smoking status, alcohol consumption, and family history of renal cancer, and for listed metabolite

**Table 5:** Attenuation of the odds ratios for the metabolite–renal cell carcinoma association with the addition of BMI to the adjustment set.

Metabolite	Metabolite OR (95% CI) * Not BMI-Adjusted	Metabolite OR (95% CI) * BMI-Adjusted	Attenuation of Log (Metabolite OR)
C38:4 PI	0.35 (0.21–0.61)	0.31 (0.18–0.56)	−11.6%
C34:0 PC	0.43 (0.26–0.72)	0.41 (0.24–0.69)	−5.6%
C3-DC-CH3 Carnitine	2.83 (1.73–4.64)	2.49 (1.49–4.17)	12.3%
C5 Carnitine	2.88 (1.74–4.76)	2.41 (1.44–4.05)	16.8%
C14:0 SM	0.45 (0.27–0.73)	0.39 (0.23–0.65)	−14.7%
C16:1 SM	0.40 (0.23–0.71)	0.33 (0.18–0.60)	−21.0%

* ORs correspond to RCC risk of 90th percentile of metabolite level compared to the 10th percentile. Model was adjusted for age, sex, race, cigarette smoking status, alcohol consumption, and family history of renal cancer.

## Data Availability

Data presented in this study available upon proposal submission to the PLCO Cancer Screening Trial, per terms of participant consent.

## Supplementary Materials

The following supporting information can be downloaded at: https://www.mdpi.com/article/10.3390/metabo12121189/s1, Table S1: Full description of metabolomics lab methodologies, Table S2: Results for assessing non-linearity of the association between renal cell carcinoma and metabolite levels, for metabolites where false discovery rate < 0.20 from conditional logistic regression; Table S3: Odds ratios (ORs) and 95% confidence intervals (CIs) for renal cell carcinoma comparing the 90th and 10th percentiles, based on the distribution in controls; Table S4: Correlations between metabolites associated with renal cell carcinoma risk and other renal cell carcinoma risk factors; Supplemental Data.

## Abbreviations

BMI: body mass index; CI: confidence interval; GGM: Gaussian graphical model; ICC: intraclass correlation; IQR: interquartile range; LC: liquid chromatography; MS: mass spectrometry; OR: odds ratio; PLCO: Prostate, Lung, Colorectal, and Ovarian Cancer Screening Trial; RCC: renal cell carcinoma; US: United States.
