# Supplementary material for: Metabolomic Analysis of Renal Cell Carcinoma in the Prostate, Lung, Colorectal, and Ovarian Cancer Screening Trial
Source: Metabolites. 2022 Nov 29;12(12):1189. doi: 10.3390/metabo12121189 (PMC9785244; doi:10.3390/metabo12121189)
Supplement: Supplementary file 1 [file metabolites-12-01189-s001.zip › PLCO RCC and Metabolomics Supplemental Tables 091722 FINAL.pdf]

Supplemental Table S1. Full description of metabolomics lab methodologies

Lab	Metabolomics Methodologies
**Broad Institute (Clary Clish lab)**	Polar metabolites and lipids in plasma extracts were measured with liquid chromatography–mass spectroscopy (LC–MS) methods. Lipids were analyzed using a Nexera X2 U-HPLC (Shimadzu, Marlborough, MA) coupled to an Exactive Plus Orbitrap mass spectrometer (Thermo Fisher Scientific; Waltham, MA). Plasma extracts (2 µL) were injected onto an ACQUITY UPLC BEH C8 column (1.7 µm, 2.1 × 100 mm; Waters; Milford, MA). The column was initially eluted isocratically at a flow rate of 450 µL/min with 80% mobile phase A (95:5:0.1 vol/vol/vol 10 mM ammonium acetate/methanol/formic acid) for one minute followed by a linear gradient to 80% mobile phase B (99.9:0.1 vol/vol methanol/formic acid) over two minutes, a linear gradient to 100% mobile phase B over seven minutes, then three minutes at 100% mobile phase B. MS analyses used electrospray ionization in the positive-ion mode (source voltage was 3 kV, source temperature was 300°C, sheath gas was 50.0, auxiliary gas was 15) and full-scan analysis over m/z 200–1100 and at 70,000 resolution. Hydrophilic interaction liquid chromatography (HILIC) analyses of water-soluble metabolites were conducted in the positive-ion mode using a Nexera X2 U-HPLC (Shimadzu, Marlborough, MA)-Q Exactive Orbitrap (Thermo Fisher Scientific; Waltham, MA) LC–MS instrument. Plasma samples (10 µL) were extracted using 90 µL of 74.9:24.9:0.2 vol/vol/vol acetonitrile/methanol/formic acid containing stable isotope-labeled internal standards (valine-d8, Isotec; and phenylalanine-d8, Cambridge Isotope Laboratories; Andover, MA). Samples were centrifuged (10 minutes, 9,000g, 4°C), and the supernatants were injected directly onto a 150 × 2 mm Atlantis HILIC column (Waters; Milford, MA). The column was eluted isocratically at a flow rate of 250 µL/min with 5% mobile phase A (10 mM ammonium formate and 0.1% formic acid in water) for one minute followed by a linear gradient to 40% mobile phase B (acetonitrile with 0.1% formic acid) over ten minutes. The electrospray ionization voltage was 3.5 kV and data were acquired using full-scan analysis over m/z 70–800 at 70,000 resolution. Progenesis CoMet (Nonlinear Dynamics) and TraceFinder 3.1 (Thermo Fisher Scientific) software were used to integrate LC–MS peaks in data generated using Orbitrap MS systems. Chromatographic retention times were determined from reference standards and reference samples for each annotated metabolite.

**Robert Gerszten lab (Massachusetts General Hospital)**	Central metabolites including sugars, sugar phosphates, organic acids, purine, and pyrimidines, are extracted from 30 µL of plasma using acetonitrile and methanol and separated using a 100 x 2.1 mm XBridge Amide column (Waters; Milford, MA). A high sensitivity Agilent 6490 QQQ MS (Agilent Technologies, Inc.; Santa Clara, CA) is used to profile metabolites in the negative ion mode via multiple reaction monitoring (MRM) scanning. MRM parameters for approximately 200 metabolites have been optimized by infusing authentic reference standards. Raw data are processed using MassHunter Quantitative Analysis Software (Agilent Technologies, Inc.; Santa Clara, CA).<br>Raw data were standardized to mitigate temporal drift in MS instrument sensitivity by scaling data, using a “nearest neighbor” approach, to pooled plasma reference samples that were inserted in the analysis queue at regular intervals of approximately 20 study samples

Supplemental Table S2. Results for assessing non-linearity of the association between renal cell carcinoma and metabolite levels, for metabolites where false discovery rate <0.20 from conditional logistic regression

Metabolite	*p* for Curvature*
**Glycerophospholipids**	
C38:4 PI	0.57
C34:0 PC	0.94
**Fatty acyls (acylcarnitines)**	
C3-DC-CH3 Carnitine	0.82
C5 Carnitine	0.61
**Sphingolipids**	
C14:0 SM	0.66
**Organic nitrogen compounds**	
C16:1 SM	0.28

*Model was adjusted for age, sex, race, cigarette smoking status, alcohol consumption, family history of renal cancer, body mass index, physical activity, history of diabetes, and history of hypertension

Supplemental Table S3. Odds ratios (ORs) and 95% confidence intervals (CIs) for renal cell carcinoma comparing the 90th and 10th percentiles, based on the distribution in controls

Metabolite	Model 1*<br>OR† (95% CI)	Model 2‡<br>OR† (95% CI)
C3-DC-CH3 Carnitine§	2.83<br>(1.73-4.64)	2.61<br>(0.53-4.47)
C5 Carnitine§	2.88<br>(1.74-4.76)	2.31<br>(1.36-3.93)
C38:4 PI§	0.35<br>(0.21-0.61)	0.32<br>(0.18-0.58)
Glutamate	3.26<br>(1.75-6.06)	2.48<br>(1.25-4.89)
C3-DC Carnitine	2.61<br>(1.55-4.40)	2.35<br>(1.34-4.10)
C22:1 SM	0.40<br>(0.23-0.69)	0.44<br>(0.25-0.77)
C34:0 PC§	0.43<br>(0.26-0.72)	0.43<br>(0.26-0.74)
Quinolinic acid	2.07<br>(1.33-3.22)	1.70<br>(1.06-2.73)
C22:0 LPE	0.42<br>(0.25-0.71)	0.52<br>(0.29-0.91)
C14:0 SM§	0.45<br>(0.27-0.73)	0.40<br>(0.24-0.68)
C40:10 PC	0.43<br>(0.26-0.73)	0.53<br>(0.31-0.92)
C16:1 SM§	0.40<br>(0.23-0.71)	0.34<br>(0.19-0.63)
DMGV	2.09<br>(1.32-3.30)	1.68<br>(1.01-2.79)
C22:6 LPE	0.44<br>(0.26-0.75)	0.53<br>(0.31-0.91)
C36:0 PC	0.46<br>(0.28-0.76)	0.46<br>(0.27-0.78)
C34:2 PC plasmalogen	0.46<br>(0.285-0.77)	0.55<br>(0.32-0.96)
Putative uridine/Pseudouridine 3	3.91<br>(1.56-9.81)	3.04<br>(1.16-7.96)
C40:9 PC	0.44<br>(0.25-0.77)	0.50<br>(0.28-0.90)
C38:6 PC	0.43<br>(0.25-0.77)	0.49<br>(0.27-0.90)

C20:0 SM	0.49 (0.30-0.80)	0.52 (0.31-0.88)
C4 Carnitine	1.89 (1.22-2.95)	1.67 (1.04-2.68)
Glutamic acid	2.23 (1.27-9.93)	1.66 (0.90-3.05)
C36:4 PC B	0.50 (0.31-0.82)	0.51 (0.30-0.87)
Uracil	1.94 (1.22-3.11)	1.92 (1.18-3.13)
Putative uridine/Pseudouridine 2	6.23 (1.71-22.71)	4.05 (1.00-16.42)
Creatinine	2.09 (1.24-3.53)	1.89 (0.08-3.30)
C18:1 CE + NH4	0.49 (0.29-0.81)	0.55 (0.32-0.95)
C34:0 PS	0.48 (0.29-0.81)	0.50 (0.28-0.88)
C38:2 PE	0.50 (0.31-0.82)	0.56 (0.34-0.94)
C36:1 PS plasmalogen	0.50 (0.30-0.82)	0.47 (0.27-0.80)
C16:0 SM	0.50 (0.30-0.82)	0.54 (0.32-0.92)
C22:5 CE + NH4	0.50 (0.31-0.83)	0.57 (0.34-0.97)
α-Ketoglutaric acid	1.79 (1.17-2.74)	1.59 (1.03-2.47)
3-Methoxytyramine	1.67 (1.15-2.43)	1.64 (1.10-2.43)
Dimethylglycine	2.04 (1.21-3.42)	1.76 (1.01-3.08)
1-Methyladenosine	2.02 (1.21-3.38)	1.50 (0.87-2.61)
C16:0 CE + NH4	0.49 (0.29-0.82)	0.54 (0.31-0.95)
N6-Acetyllysine	1.88 (1.17-3.00)	1.83 (1.10-3.04)
C34:4 PC	0.50 (0.29-0.84)	0.49 (0.28-0.87)
C36:0 PE	0.52 (0.32-0.86)	0.52 (0.31-0.89)
C18:0 LPE	0.53 (0.33-0.86)	0.59 (0.36-0.99)
21-Deoxycortisol	0.60 (0.41-0.89)	0.63 (0.42-0.96)

C20:4 LPE	0.52 (0.31-0.86)	0.60 (0.35-1.02)
Urate	1.92 (1.16-3.18)	1.50 (0.85-2.64)
Choline	2.03 (1.18-3.50)	1.73 (0.96-3.14)
C16:1 LPC plasmalogen	0.51 (0.30-0.86)	0.56 (0.32-0.98)
Pyruvic acid	1.87 (1.15-3.06)	1.80 (1.06-3.08)
S-Adenosyl-L-homocysteine	1.94 (1.16-3.25)	1.65 (1.06-2.56)
C16:0 LPE	0.54 (0.33-0.88)	0.66 (0.39-1.11)
C34:3 PC plasmalogen	0.55 (0.64-0.88)	0.63 (0.38-1.05)
C24:0 SM	0.54 (0.33-0.89)	0.59 (0.36-0.99)
2,3-O-Methyladenosine	2.17 (1.16-4.07)	1.88 (0.96-3.67)
Isoleucine	1.82 (1.12-2.98)	1.44 (0.83-2.51)
Uric acid	1.75 (1.11-2.77)	1.54 (0.98-2.40)
6,8-Dihydroxypurine	1.63 (1.09-2.44)	1.44 (0.93-2.21)
C20:0 LPE	0.53 (0.32-0.90)	0.66 (0.38-1.15)
C18:0 LPC	0.52 (0.30-0.90)	0.54 (0.30-0.96)
LPE Source fragment	1.72 (1.09-2.72)	1.72 (1.05-2.83)
C32:0 PC	0.56 (0.34-0.92)	0.60 (0.35-1.03)
Hypoxanthine	1.80 (1.09-2.97)	1.84 (1.08-3.15)
C34:1 PC	0.57 (0.35-0.92)	0.57 (0.34-0.96)
C18:2 CE + NH4	0.56 (0.34-0.92)	0.63 (0.37-1.06)
C20:3 LPC	0.56 (0.34-0.92)	0.63 (0.37-1.07)
C16:0 LPC	0.56 (0.34-0.92)	0.63 (0.37-1.07)
C32:2 PC	0.55 (0.33-0.92)	0.52 (0.30-0.91)

C24:0 Ceramide D18:1	0.58 (0.37-0.93)	0.56 (0.34-0.93)
C22:6 LPC	0.56 (0.34-0.93)	0.68 (0.40-1.18)
Malic acid	1.70 (1.07-2.70)	1.84 (1.13-3.00)
Valine	1.77 (1.07-2.92)	1.35 (0.76-2.39)
Phenylalanine	1.71 (1.07-2.74)	1.47 (0.88-2.46)
N-Carbamoyl-β-alanine	1.68 (1.06-2.66)	1.34 (0.82-2.20)
C20:5 CE + NH4	0.58 (0.35-0.94)	0.57 (0.34-0.97)
C20:4 CE + NH4	0.58 (0.35-0.94)	0.61 (0.36-1.03)
C34:0 PE	0.57 (0.34-0.94)	0.55 (0.32-0.94)
C5:1 Carnitine	1.69 (1.06-2.70)	1.55 (0.95-2.55)
Anserine	1.65 (1.05-2.59)	1.56 (0.97-2.50)
Carnitine	1.71 (1.06-2.77)	1.42 (0.84-2.40)
C3 Carnitine	1.69 (1.05-2.72)	1.30 (0.77-2.18)
C22:0 SM	0.58 (0.35-0.95)	0.61 (0.36-1.03)
C30:0 PC	0.57 (0.34-0.95)	0.53 (0.31-0.91)
C20:5 LPC	0.56 (0.33-0.95)	0.70 (0.40-1.23)
Cyclic AMP	0.51 (0.28-0.94)	0.43 (0.22-0.84)

Metabolites presented here were significant at the false discovery rate <0.20 for model 1

*Model was adjusted for age, sex, race, cigarette smoking status, alcohol consumption, and family history of renal cancer

†ORs correspond to RCC risk at the 90th percentile compared to the 10th percentile of log metabolite intensity

‡Model was adjusted for model 1 covariates, plus body mass index, physical activity, history of diabetes, and history of hypertension

§Metabolite was also significant at the false discovery rate <0.20 for model 2

Supplemental Table S4. Correlations between metabolites associated with renal cell carcinoma risk and other renal cell carcinoma risk factors

Risk Factor	C38:4 PI	C34:0 PC	C3-DC-CH3 Carnitine	C5 Carnitine	C14:0 SM	C16:1 SM
**Age**	-0.06	0.03	0.16	-0.001	-0.05	-0.03
**Sex**	0.17	0.01	-0.06	-0.27	0.25	0.38
**Race**	0.03	0.01	-0.13	-0.03	-0.17	-0.04
**BMI**	0.10	-0.06	0.15	0.18	0.10	0.10
**Physical activity**	-0.06	0.07	-0.05	-0.07	-0.05	-0.03
**History of diabetes**	-0.01	-0.06	0.03	0.11	-0.11	-0.12
**History of hypertension**	0.003	-0.06	0.10	0.13	-0.09	-0.05
**Cigarette smoking status**	-0.07	0.02	-0.01	0.06	-0.02	-0.01
**Alcohol consumption**	0.001	0.17	-0.08	0.02	0.09	0.02
**Family history of renal cancer**	-0.02	0.003	-0.02	-0.04	0.0001	0.02
**C38:4 PI**	--	0.59	-0.009	-0.06	0.47	0.55
**C34:0 PC**	0.59	--	0.08	-0.03	0.60	0.65
**C3-DC-CH3 Carnitine**	-0.009	0.08	--	0.39	0.07	0.06
**C5 Carnitine**	-0.06	-0.03	0.39	--	-0.06	-0.17
**C14:0 SM**	0.47	0.60	0.07	-0.03	--	0.71
**C16:1 SM**	0.55	0.65	0.06	-0.17	0.71	--
